# Effect of Bone Resorption Inhibitors on Serum Cholesterol Level and Fracture Risk in Osteoporosis: Randomized Comparative Study Between Minodronic Acid and Raloxifene

**DOI:** 10.1007/s00223-023-01060-9

**Published:** 2023-01-28

**Authors:** Hiroaki Ohta, Yukari Uemura, Teruki Sone, Shiro Tanaka, Satoshi Soen, Satoshi Mori, Hiroshi Hagino, Masao Fukunaga, Toshitaka Nakamura, Hajime Orimo, Masataka Shiraki, Adequate Treatment of Osteoporosis (A-TOP) research group

**Affiliations:** 1 Department of Obstetrics and Gynecology 2, Kawasaki Medical School, Okayama, Japan; 2 Biostatistics Section, Department of Data Science, Center for Clinical Sciences, National Center for Global Health and Medicine, 1-21-1, Toyama, Shinjyuku-Ku, Tokyo, 162-8655 Japan; 3 Department of Nuclear Medicine, Kawasaki Medical School, Okayama, Japan; 4 Department of Clinical Biostatistics, Graduate School of Medicine, Kyoto University, Kyoto, Japan; 5 Soen Orthopaedics, Osteoporosis and Rheumatology Clinic, Hyogo, Japan; 6 Bone and Joint Surgery, Seirei Hamamatu General Hospital, Shizuoka, Japan; 7 School of Health Science, Tottori University Faculty of Medicine, Tottori, Japan; 8 Kawasaki Medical School, Okayama, Japan; 9 Touto Sangenjaya Rehabilitation Hospital, Tokyo, Japan; 10 Japan Osteoporosis Foundation, Tokyo, Japan; 11 Department of Internal Medicine, Research Institute and Practice for Involutional Diseases, Nagano, Japan

**Keywords:** Cholesterol, Nitrogen-containing bisphosphonates, Selective estrogen receptor modulators, Fractures, Osteoporosis

## Abstract

The positive link between osteoporosis and hypercholesterolemia has been documented, and bone resorption inhibitors, such as nitrogen-containing bisphosphonates (N-BP) and selective estrogen receptor modulators (SERMs), are known to reduce serum cholesterol levels. However, the relationship between the baseline cholesterol level and incident fracture rate under the treatment using the bone resorption inhibitors has not been documented. We investigated the relation between vertebral fracture incident and the baseline cholesterol levels and cholesterol-lowering effect of N-BP and SERM in osteoporosis through a prospective randomized open-label study design. Patients with osteoporosis (*n* = 3986) were allocated into two groups based on the drug used for treatment: minodronic acid (MIN) (*n* = 1624) as an N-BP and raloxifene (RLX) as an SERM (*n* = 1623). Serum levels of cholesterol and incidence of vertebral fracture were monitored for 2 years. The vertebral fracture rates between the two groups were compared using the pre-specified stratification factors. The patients receiving MIN with baseline low-density lipoprotein (LDL)-cholesterol level of ≥ 140 mg/dL, high-density lipoprotein cholesterol level < 40 mg/dL, age group of ≥ 75 years, and T score of BMD ≥ -3 SD had significantly lower vertebral fracture rates than those receiving RLX (incidence rate ratios (IRR) 0.45 [95% confidence interval (CI) 0.30 0.75, *p* = 0.001], 0.25 [95% CI 0.09 0.65, *p* = 0.005], 0.71 [95% CI 0.56 0.91, *p* = 0.006], 0.47 [95% CI 0.30 0.75, *p* = 0.0012], respectively). The cholesterol-lowering effect was stronger in the RLX group than in the MIN group, regardless of prior statin use. These results indicated that MIN treatment was more effective in reducing fracture risk in patients with higher LDL cholesterol levels, although its cholesterol-lowering ability was lesser than the RLX treatment.

*Trial registration* University Hospital Medical Information Network-Clinical Trials Registry (UMIN-CTR), No. UMIN000005433; date: April 13, 2011.

## Introduction

Life expectancy has increased worldwide, and this situation has enhanced the susceptibility to involutional diseases, such as cardiovascular disease (CVD), osteoporotic fractures, malignant tumors, and cognitive impairment. These morbid conditions account for a significant socio-economic burden, especially in an aging society. Many epidemiological studies have indicated a positive link between CVD and osteoporotic fracture or low bone mineral density (BMD) and high low-density lipoprotein cholesterol (LDL-C) or total cholesterol level [1–5]. Moreover, cholesterol-lowering statins increase BMD and reduce the incidence of osteoporotic fracture [6, 7]. These evidence suggest that the cholesterol metabolism and bone health may have a tight link and improvement in lipid metabolism may provide better bone health in osteoporosis through the modifications of bone cell functions [8]. However, there has been contradictions in the relationship between serum cholesterol level and fracture risk [9, 10]. Therefore, the further investigation of the relationship between the baseline serum cholesterol level and fracture incidence in osteoporotic patients under treatment may be required.

Nitrogen-containing bisphosphonates (N-BPs) inhibit the mevalonate pathway, which is the main target metabolic process of statins, and this inhibitory effect is responsible for the inhibition of bone resorption [11]. N-BP selectively and potently inhibits farnesyl diphosphate synthase [12]. In contrast, statins inhibit β-hydroxy β-methylglutaryl-CoA (HMG-CoA) reductase, which is positioned upstream of farnesyl pyrophosphate (FPP) synthase in the mevalonate pathway. This evidence indicates that both N-BP and statin target the different enzymes of the mevalonate pathway and reduce cholesterol production. However, orally administered bisphosphonate is absorbed from the intestine, and approximately, 50% of the absorbed drug is selectively retained in the skeleton, whereas the remainder is quickly eliminated into the urine without being metabolized [13], suggesting that the effect of N-BP on cholesterol metabolism may be limited in bone, but not in other organs. However, the high affinity of N-BP for bone mineral allows bisphosphonates to achieve a high local concentration throughout the skeleton. Although FPP synthase is ubiquitously expressed in mammalian cells and plays a critical role in cholesterol production, cellular apoptosis induced by N-BP appears to occur only in osteoclasts [13].

Adami et al*.* reported that N-BP (neridronate) decreased LDL-C and increased high-density lipoprotein cholesterol (HDL-C) [14]. In contrast to N-BP, selective estrogen receptor modulators (SERMs) have been reported to have cholesterol-lowering effects through modulation of lipoprotein metabolism in the liver, similar to estrogen [15]. This cholesterol-lowering effect of SERMs has been confirmed in a clinical trial [16]. Therefore, both N-BP and SERM are expected to have clinical utility in cholesterol metabolism in osteoporosis.

However, until now, there have been no robust data that suggest an osteoporotic drug that has a more beneficial effect on cholesterol metabolism, thereby having additional health benefits. Therefore, it may be important to investigate which class of bone resorption inhibitor has a stronger cholesterol-lowering effect and whether the effect on the cholesterol metabolism is associated with fracture risk reduction or not.

The aim of the present study was to clarify the relationship between the baseline cholesterol level and incident fracture rate under N-BP and SERM. In addition, we investigated the effects of N-BP and SERM on serum cholesterol levels using a randomized open-label prospective study design [17, 18]. This study was carried out as a secondary end point of the JOINT 04 study [17, 18], which was conducted to compare the fracture prevention ability between an N-BP (minodronate: MIN) and a SERM (raloxifene: RLX) in postmenopausal women with osteoporosis. This study is the first report to clarify the effect of bone resorption inhibitors on cholesterol metabolism in relation to fracture risk reduction in osteoporosis.

## Methods

### Study Design

The details of the study protocol (JOINT 04 protocol) have been reported elsewhere [17, 18]. In brief, subjects who met all study entry criteria were enrolled and randomized in a ratio of 1:1 to receive minodronate (MIN) as an N-BP or raloxifene (RLX) as an SERM. Subjects allocated to the RLX group were treated with oral doses of 60 mg/day, whereas those allocated to the MIN group received doses of either 1 mg/day or 50 mg/4 weeks. Randomization was implemented using a web-based computerized system with the modified minimization method that adjusted imbalances in six variables, as defined by the Japanese guidelines for the prevention and treatment of osteoporosis (2006 edition) [19]. Imbalance of the following predefined variables was to be avoided among the groups: age; number of pre-existing vertebral fractures; history of non-vertebral fractures of the humerus, femur, or radius; BMD; number of risk factors (alcohol intake, smoking, and history of parents’ femoral neck fractures); and study sites. The treatment period was 2 years.

### Study Endpoint

The primary endpoint of the JOINT 04 protocol [18] was fracture risk reduction between the MIN and RLX groups. The results of the primary endpoint have been reported elsewhere [18]. In the present study, a secondary analysis was performed to compare the changes in cholesterol levels between the two groups. The relationship between the baseline cholesterol levels and fracture risk reduction in the two arms was also investigated.

### Study Participants

The inclusion criteria were as follows: women aged ≥ 60 years or older and could walk by themselves; those who could answer questionnaires; and those who could satisfy the criteria to start pharmacotherapy, which were defined by the Japanese guidelines for the prevention and treatment of osteoporosis (2006 edition) [19]. In addition, one of the following risk factors for incident fractures needed to be relevant to each of the participants: age ≥ 70 years, one or more prevalent fractures at the vertebrae (Th4 to L4), and BMD < − 3 SD of the young adult mean (YAM). Subjects were excluded if they had contraindications to the test drugs, the presence of metabolic bone diseases other than osteoporosis, severe degenerative deformation of the spine (T4–L4), and the presence of critical illness at the time of registration. The subjects undergoing pretreatment with RLX or MIN required washout of the treatment (1 month prior for RLX and 6 months prior for MIN). For patients under statin treatment, statin use was continued during the study period.

### Treatment Protocol

As indicated above, the test drugs were randomly allocated to the participant, and the allocated treatment was continued for 2 years. If the patient had been receiving statins or other cholesterol-lowering drugs before registration, the drug use was continued during the test period.

### Assessment of Efficacy

The assessment of prevalent and incident fractures has been reported previously [17, 18]. The assessments of the fractures were carried out by the independent central committee to mask the patient’s information. The fracture was assessed before and at 6, 12, and 24 months of treatment. In addition, X-ray evaluation was performed on demand [20]. Non-fasting serum levels of total cholesterol (TC), LDL-C, HDL-C, and triglycerides were measured before treatment and at 6 and 12 months after treatment. The samples were measured by LSI Medience Corporation (Tokyo, Japan). Also, in the present study, subjects were stratified using the pre-specified criteria for age (75 years), BMD (-3SD), BMI (25 kg/m^2^), serum levels of LDL-C of 140 mg/dL (3.6 nmole/L), HDL-C of 40 mg/dL (1.03 nmole/L), and triglycerides of 150 mg/dl (1.69 nmole/L). The thresholds for LDL and HDL-C are in accordance with the Japanese criteria of hyperlipidemia [21].

### Statistical Analyses

Continuous variables were expressed by the mean** ± **standard deviation (SD) or 95% confidence interval (CI). To analyze the lipid-lowering effect of the test drugs, the changes in the TC, LDL-C, HDL-C, and triglyceride (TG) levels during the treatment were compared between the MIN and RLX groups. Differences between the two arms at each visit were compared using *t* tests. The differences between the baseline and follow-up values were compared between the two groups. These values were also compared among the subgroups with or without statin use. The incident vertebral fracture rate was analyzed in the subgroups with or without the pre-specified fracture risk expressed by the forest plot. Furthermore, the relationship between the baseline lipid values and fracture incidence was analyzed using a logistic regression model with a spline curve after adjustment for age, BMD, and number of prevalent fractures, which were considered to be major confounder for incident fracture risk. Statistical analysis was performed using SAS version 9.4 (SAS Institute, Cary, NC, USA).

### Study Oversight

This study was registered at the University Hospital Medical Information Network-Clinical Trials Registry (UMIN-CTR) under the identification number UMIN000005433. The date of the registration was April 13, 2011. The protocol was approved by the Central Ethical Committee for Adequate Treatment of Osteoporosis group (Chairman Dr. Rikushi Morita) and was reviewed by the institutional review board of each participating institution. The trial was conducted in accordance with the principles of the Declaration of Helsinki. Written informed consent was obtained prior to patient enrollment after a thorough explanation of the trial objectives, duration, and procedures.

## Results

### Subjects

We randomized 3986 patients from 266 sites nationwide. The final analysis was performed for 3247 cases. These patients were randomly assigned to the MIN (n = 1623) or RLX arm (*n* = 1624). The mean and SD range of age in the total cases was 75.2 $\pm$ 6.7 years. The number of patients who discontinued the allocated treatment was similar between the two groups [18]. There was no statistical difference in the background characteristics between the two groups, as indicated in Table 1, suggesting no obvious selection bias. A total of 745 patients were reported to be treated with statins (368 for MIN group and 377 for RLX group) at baseline, and the treatment was continued during the study period. No statistical difference in the prevalence of statin users was observed in either group.

**Table 1:** Background data (full analysis set: *n* = 3247)

	Minodronate (*N* = 1623)		Raloxifene (*N* = 1624)	
	Mean or *N*	SD or %	Mean or *N*	SD or %
Age, years	75.3	6.7	75.1	6.7
Height, cm	148.7	6.3	148.5	6.2
Weight, kg	50.2	8.7	50.3	8.4
Total cholesterol, mg/dL	207.2	35.8	207.5	33.2
LDL-C*, mg/dL	118.3	29.9	118.5	28.1
LDL-C ≥ 140 mg/dL	375	23.1	341	21.0
HDL-C*, mg/dL	61.8	15.4	62.2	15.5
HDL ≥ 40 mg/dL	1541	94.9	1538	94.7
Triglycerides, mg/dL	130.4	69.6	127.8	64.9
Triglycerides ≥ 150 mg/dL	465	28.7	445	27.4
HbA1c, %	5.9	0.6	5.9	0.6
Hypertension**	862	53.1%	842	51.8%
Diabetes mellitus**	197	12.1%	173	10.7%
Dyslipidemia**	512	31.5%	532	32.8%
Alcohol drinker	38	2.3%	35	2.2%
Smoker	71	4.4%	61	3.8%
Statin user	368	22.6%	377	23.2%

*LDL-C and HDL-C mean low- and high-density lipoprotein cholesterol, respectively

**Hypertension, diabetes mellitus, and dyslipidemia were diagnosed by the corresponding diagnostic criteria. All the items listed in the table were not significantly different, suggesting that the background characteristics were well-balanced distributed

### Fracture Analysis in Relation to the Serum Levels of Lipid

**Fig. 1:**
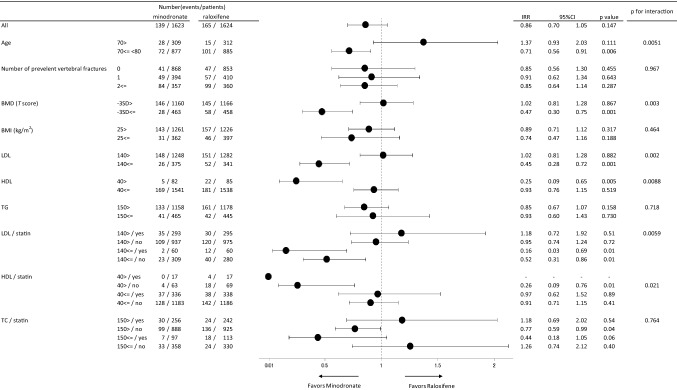
Subgroup analysis of the effects of treatment on vertebral fractures according to pre-specified subgroups. The forest plot of the minodronic acid (MIN) and raloxifene (RLX) treatments on the vertebral fracture incidence among the pre-specified fracture risk. The overall difference in incident vertebral fracture rate between MIN and RLX was not significant, but the subgroups of pre-specified background indicated that the MIN treatment showed a significant reduction in fracture risk compared to the RLX treatment, such as age ≥ 75 years (*p* = 0.0051), *T* score of BMD ≥ -3SD (*p* = 0.0035), LDL-C ≥ 140 mg/dL (*p* = 0.0020), and HDL-C < 40 mg/dL (*p* = 0.0083). *MIN* minodronic acid; *RLX* raloxifene; *LDL-C* low-density lipoprotein cholesterol; *HDL-C* high-density lipoprotein cholesterol

Figure 1 shows the forest plot of incident vertebral fractures for a total of seven pre-specified stratification factors. Among them, the incident vertebral fracture rate of the MIN arm in four factors, such as age group of ≥ 75 years, *T* score of BMD ≥ -3 SD, LDL-C ≥ 140 mg/dL, and HDL-C < 40 mg/dLs, was lower than that in the corresponding group of the RLX arm. Incident rate ratios (IRR) for the MIN group against the RLX group in each pre-specified parameter were 0.71 (95% CI 0.56–0.91, *p* = 0.006), 0.47 (95% CI 0.30–0.75, *p* = 0.001), 0.45 (95% CI 0.28–0.72, *p* = 0.001), and 0.25 (95% CI 0.09–0.65, *p* = 0.005), respectively. The incident vertebral fracture rates for LDL < 140 mg/dL were lower in the MIN group compared to the RLX group, irrespective of the statin use prior to the start of the observation. Also, the interaction effects between the four factors of age, BMD, LDL, and HDL and the treatment group were evaluated simultaneously after adjusting for the important confounding factors. The p values for the interaction were estimated to be 0.047, 0.066, 0.12, and 0.24, respectively.

**Fig. 2:**
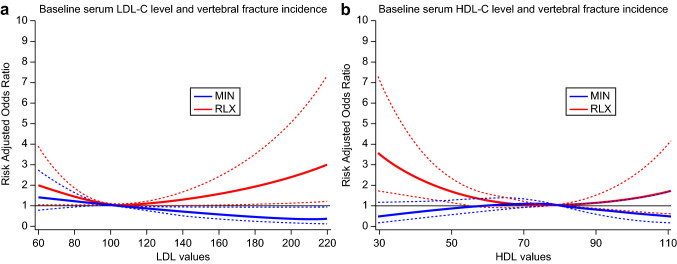
Relationship between serum cholesterol levels and vertebral fracture incidence indicated by odds ratios after adjustment of age, BMD, and the number of prevalent vertebral fracture. The lines are depicted by spline curve of odds ratios. Bold and thin lines indicate MIN- and RLX-treated groups, respectively. The RLX-treated group showed higher odds ratio for incident vertebral fracture than those in MIN-treated group having higher LDL-C or lower HDL-C levels. The dotted lines indicated the 95% confidence intervals for each treatment arm. *MIN* minodronic acid; *RLX* raloxifene; *LDL-C* low-density lipoprotein cholesterol; *HDL-C* high-density lipoprotein cholesterol; *BMD* bone mineral density

The association between the baseline serum levels and vertebral fracture incidence was estimated as IRR 0.94 [95% CI 0.74 1.22, *p* = 0.67] and IRR 1.52 [95% CI 1.02 2.27, *p* = 0.041], for lower LDL (LDL-C < 140 mg/dL) and HDL-C (HDL-C < 40 mg/dL). However, the trend between the baseline serum levels and vertebral fracture incidence was not identical among the treatment groups as shown in Fig. 2a and b. Figure 2a depicts the odds ratios, showing the association of the baseline LDL-C level with the reference of 100 mg/dL and the vertebral fracture incidence, for both minodronate and raloxifene group. Similarly, Fig. 2b shows the odds ratios for HDL-C level with the reference of 80 mg/dL. The odds ratios were estimated using the logistic regression model, and the association was expressed by the spline curve after adjustment for age, BMD, and number of prevalent vertebral fractures. The trend between the baseline LDL-C and vertebral fracture incidence significantly differed between the treatment groups (*p* = 0.0129), also the trend for the baseline HDL-C differed significantly (*p* = 0.047). The fracture risk reduction in the MIN group (blue line) tended to be superior to that in the RLX group (red line) in accordance with baseline LDL- and HDL-C levels. The dotted blue and red lines indicated the 95% confidence interval line for MIN and RLX treatment, respectively.

### Lipid Analysis

**Fig. 3:**
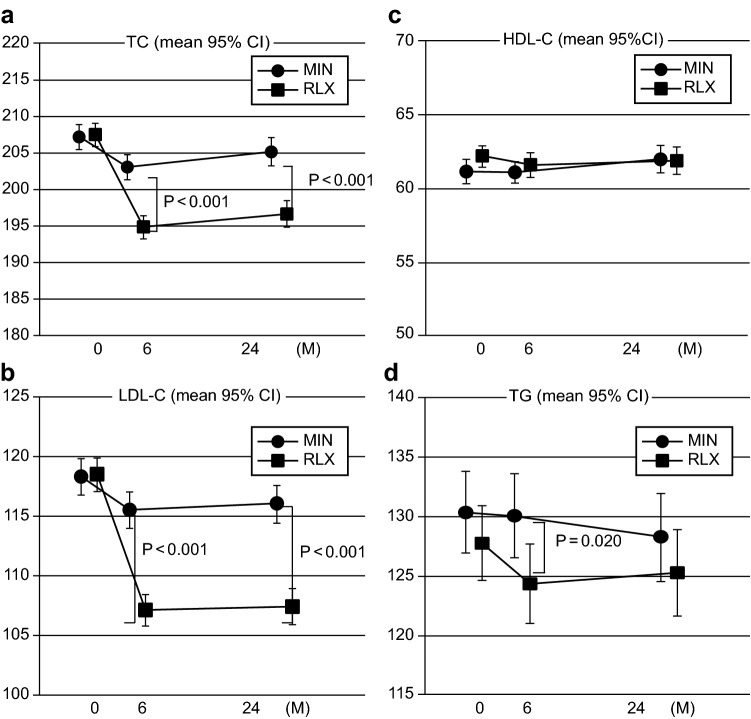
Changes in serum levels of lipid markers before and after the treatment. Both treatment groups showed significant decline in lipid marker levels except for serum level of HDL-C. The RLX-treated group showed significantly larger decline in TC, LDL-C, and TG than the MIN-treated group. *MIN* minodronic acid; *RLX* raloxifene; *LDL-C* low-density lipoprotein cholesterol; *HDL-C* high-density lipoprotein cholesterol; *TC* total cholesterol; *TG* triglyceride

The changes in serum lipid metabolites before and after the treatment are shown in Fig. 3a–d. The mean and SD range of TC at baseline and at 6 and 24 months were 207.2 ± 35.8 (mean ± SD), 203.1 ± 34.4, and 205.2 ± 34.7 mg/dL, respectively, in the MIN group (closed circle) (Fig. 3a); while those in the RLX group were 207.5 ± 32.2, 194.9 ± 30.9, and 196.7 ± 31.4 mg/dL, respectively (closed square). The TC levels in the RLX-treated group after treatment were significantly lower than those in the MIN group (*p* < 0.001). The mean and SD range of LDL-C levels in the RLX-treated group were 118.5 ± 28.1, 107.1 ± 26.2, and 107.4 ± 26.2 mg/dL for the baseline, at 6 months, and at 24 months, respectively. The values after the RLX treatment were significantly lower than the baseline value (*p* < 0.001) and the corresponding values in the MIN group (118.3 ± 29.9, 115.5 ± 29.0, and 116.0 ± 29.4 mg/dL, respectively, *p* < 0.001 versus RLX group) (Fig. 3b). Although the LDL-C levels in the MIN-treated group were significantly decreased compared to the baseline value (*p* < 0.001), the magnitude of the decline was very small. On the other hand, serum HDL-C levels in both groups did not show any significant differences among the groups and at every time point tested (Fig. 3c). The serum levels of triglycerides were statistically different only at 6 months. The corresponding values at 6 months after the initiation of the treatment was 130.1 ± 69.5 mg/dL for MIN group and 124.4 ± 66.0 mg/dL for RLX group (*p* = 0.020). (Fig. 3d). In the present study, the incidence rate of vascular accident in both groups did not show any difference between MIN and RLX treatment groups, although the lipid profile in RLX group were superior to those in MIN group, probably because of the shorter observation period and/or lower lipid level at baseline.

**Fig. 4:**
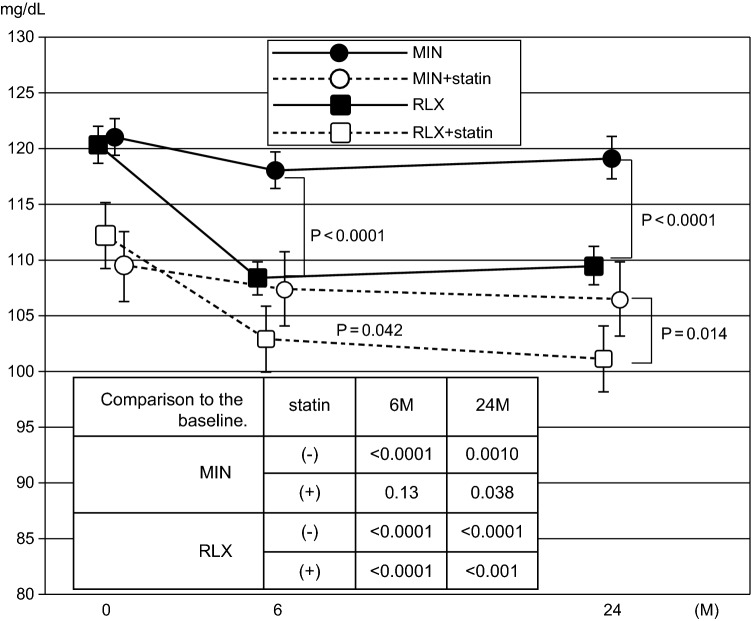
Changes in serum level of LDL cholesterol in the patients with or without statin use. A total of 745 patients out of 3246 participants (377 for RLX and 368 for MIN) were treated with statins prior to start the treatment of osteoporosis. The subjects were divided into four groups in accordance with the treatment received, and depending on statin use. The baseline LDL-C levels with statin users showed significantly lower baseline LDL-C levels. Serum LDL-C levels after the treatment with MIN or RLX indicated a significant decrease at 6 and 24 months treatment regardless of statin use. *MIN* minodronic acid; *RLX* raloxifene; *LDL-C* low-density lipoprotein cholesterol; *HDL-C* high-density lipoprotein cholesterol

A total of 745 out of 3246 (23%) cases (377 for RLX and 368 for MIN) were treated with statins prior to the start of the observation. There was no significant difference in concurrent statin use between the two groups. The changes in LDL-C levels in both groups, with or without statin use, are depicted in Fig. 4. The baseline LDL-C levels in both treatment groups with statin use were significantly lower than those without statin use. In addition, a further decline in LDL-C levels in statin users followed by RLX was observed. The serum LDL-C levels in the MIN group without statin use were significantly decreased from baseline at 6 (*p* < 0.001) and 24 (*p* = 0.010) months. On the other hand, the LDL-C level in the MIN group with statin use decreased slightly and showed a marginally significant decrease at only 24 months compared to the baseline value (*p* = 0.038). These data indicated that the RLX treatment resulted in a more potent decline in serum LDL-C levels regardless of whether statins were used or not, as compared to the MIN treatment.

## Discussion

It is well known that the treatment of osteoporosis using bone resorption inhibitors has health-related benefits, such as cholesterol-lowering effects, in addition to fracture risk reduction. The present study indicated that both RLX and MIN treatments reduced the serum cholesterol levels regardless of the concurrent use of statins. It has been reported that serum LDL-C level was reduced by 7–12% below the baseline value in 3 years by the RLX treatment [21]. In the present study, the serum LDL-C level decreased by 9.5% during 2 years of treatment with RLX. The decline in LDL-C levels after RLX use was therefore consistent. In the case of MIN users, the mean value of the LDL-C level decreased by approximately 2% from baseline. The cholesterol-lowering effect of N-BP (alendronate at a dose of 10 mg/day) was reported to be reduced by approximately 6% in a previous report [22]. The present study showed that the LDL-C lowering effect induced by MIN seemed to be less potent. However, the baseline LDL-C level in the previous study was 170 mg/dL, and that in the present study was 118 mg/dL. Furthermore, the dose of N-BP in Japan is approximately half of the international dose. Thus, the lower efficacy of N-BP induced cholesterol reduction in the present study compared to that of the previous report may be due to the difference in the baseline value of LDL-C and the lower dose of N-BP.

As a positive link between osteoporosis and vascular accidents has been reported previously [1–5], the cholesterol-lowering effect of bone resorption inhibitors may interfere with this link. Hence, a long-term prospective study is required to confirm this expectation. In addition, it is possible that the cholesterol-lowering effect may provide further fracture risk reduction in osteoporotic patients because high LDL-C levels have been reported to increase the incident fracture risk possibly through reduction of osteoblastic function, because oxidative products of LDL-C inhibit osteoblastic differentiation [23], which induces a reduction in bone formation. In fact, the present study indicated a higher incident fracture rate in accordance with higher LDL-C and lower HDL-C levels at baseline, even in the patients treated with RLX (Fig. 2). These results seem to contradict the cholesterol-lowering effect in the RLX and MIN groups. Although the MIN treatment showed a lower potency in terms of the cholesterol-lowering effect, the fracture risk reduction effect was stronger in patients receiving the MIN treatment than in those receiving the RLX treatment, especially in patients with higher baseline LDL-C levels. The higher fracture risk reduction in the subgroup of MIN with high baseline LDL-C levels may not be related with cholesterol-lowering effect of MIN. [18]. This finding suggested that, owing to fracture risk reduction, patients having highly reduced BMD may prefer increasing their BMD during the 2-year treatment period. The present study indicated that the higher baseline cholesterol level induced higher susceptibility of vertebral fracture but cholesterol-lowering effects of both drugs did not seem to contribute fracture risk reduction. This discrepancy may due to the term of high cholesterol exposure at baseline. The duration of high cholesterol level may be more important to fracture susceptibility but short-term lowering of cholesterol may be less potent to reduce fracture risk.

The cholesterol-lowering effect of N-BP treatment was induced by the inhibition of the mevalonate pathway through the inhibition of FPP synthase [9, 10]. On the other hand, statins inhibit HMG-CoA reductase activity, which is located upstream of FPP synthase in the mevalonate pathway. This could explain why the additive effect of MIN and statin on cholesterol level was weaker than that in the concurrent use of RLX and statin. RLX has a cholesterol-lowering effect through modulation of lipoprotein metabolism in the liver, which is similar to the effect of estrogen [16, 24–26]. Therefore, the two types of bone resorption inhibitors have cholesterol-lowering effects via different mechanisms and different potencies. The effect of concurrent use of statin and RLX on cholesterol level has been reported previously [27], and the combination treatment decreased the serum LDL-C level by 30%, while RLX or statin treatment individually decreased the serum LDL-C levels by 10% and 24%, respectively. Thus, the cholesterol-lowering effect of the concurrent use of RLX and statins in the present study was consistent.

In the present study, the LDL-C lowering effect of RLX was significantly stronger than that of MIN. As N-BP is known to be tightly accumulated into bone tissue, the cholesterol-lowering effect of N-BP may be seen in bone tissue, without systemic effects. The number of participants in the present study was the largest, resulting in sufficient statistical power; however, some study limitations were still present. First, the participants were limited to women. Therefore, the results of the present study may not be applicable to men with osteoporosis. Second, the baseline levels of the cholesterol in the present study were lower than those in the previous studies; thus, the cholesterol-lowering effect was lesser. Finally, the present study did not include patients not receiving treatment with osteoporosis because of ethical reasons.

In conclusion, both RLX and MIN treatments of osteoporosis lowered the serum cholesterol levels, especially in the RLX group. The potency of the cholesterol-lowering effect in the RLX arm was greater than that in the MIN group, and the reduction was observed regardless of whether statin was used or not. The baseline LDL-C or HDL-C levels were related to fracture risk in both groups with different patterns. The vertebral fracture risk reduction was larger in the MIN group than in the RLX group, according to the baseline LDL-C levels. The osteoporotic patients with severely decreased BMD may prefer increased BMD to reduce fracture risk than the cholesterol-lowering effect. Longer observation may be required to confirm the relationship between fracture risk reduction and cholesterol-lowering effects. The present results were the first comparative evidence of the effects between two types of bone resorption inhibitors on cholesterol metabolism in relation to fracture risk reduction.
